# Objective and Subjective Clustering Methods for Verb Fluency Responses From Individuals With Alzheimer's Dementia and Cognitively Healthy Older Adults

**DOI:** 10.1044/2023_AJSLP-22-00290

**Published:** 2023-09-18

**Authors:** Madison N. Fisher, Devin M. Casenhiser, Eun Jin Paek

**Affiliations:** a Department of Audiology and Speech Pathology, College of Health Professions, The University of Tennessee Health Science Center, Knoxville

## Abstract

**Purpose:**

The verb fluency task has been researched using a variety of analysis methods and shown its sensitivity to declines in executive functioning and lexical retrieval abilities in various neurogenic populations. Few studies to date, however, have analyzed clusters and switches in the task, and there is a lack of robust analysis methods that preclude subjectivity and potential rater bias. The purpose of this study was to investigate the reliability when using subjective clustering methods and to determine the feasibility of using an objective clustering method to determine verb fluency performance in individuals with Alzheimer's dementia (IwDs) and cognitively healthy older adults (CHOAs).

**Method:**

Responses from a verb fluency task were obtained from IwDs and CHOAs. Group differences were examined using an objective clustering method for multiple variables regarding clustering and switching. We also calculated the intrarater, interrater, and intermethod reliability using intraclass coefficients.

**Results:**

Significant group differences were found when utilizing the objective clustering method in all variables except the average cluster size, with IwDs performing poorer than CHOAs. Intrarater reliability was excellent. Interrater reliability between two authors and intermethod reliability between the objective and subjective methods were variable ranging from moderate to good.

**Conclusions:**

The results from using the objective clustering method in this study are consistent with the previous literature, making it a viable option for clustering analyses on the verb fluency task, which naturally minimizes subjectivity and rater bias. Alternatively, employing a thoroughly validated and reliable subjective approach can also mitigate potential rater bias and improve replicability across studies.

**Supplemental Material:**

https://doi.org/10.23641/asha.24061017

Verbal fluency tasks require spontaneous generation of words in a specific amount of time, typically 30 or 60 s, with a predetermined set of criteria (e.g., producing words in a semantic category such as animals). Verbal fluency tasks, including phonemic or letter fluency (producing words beginning with a specified sound or letter) and category or semantic fluency (producing words within a specific category such as animals or supermarket items), have been used to assess and monitor executive functioning and lexical access in a variety of neurogenic populations (Alegret et al., 2018; Henry et al., 2004; Macoir et al., 2019; Östberg et al., 2005; Paek & Murray, 2021; Rodrigues et al., 2015). Category fluency tasks in which the participant is asked to produce verbs of a specific semantic category (as opposed to nouns of a specific category) are a more recent addition to the set of verbal fluency tasks and were first studied by Piatt, Fields, Paolo, and Troster (1999). The “verb fluency tasks” have been shown to be valid and reliable measures of multiple domains of cognition and predictors of future cognitive declines in the assessment of cognitive–linguistic skills in adults (Macoir et al., 2019; Östberg et al., 2005; Paek & Murray, 2021; Zhao et al., 2013; to Piatt, Fields, Paolo, Koller, & Troster, 1999; Piatt, Fields, Paolo, & Troster, 1999). For example, those with neurological disorders such as aphasia or Alzheimer's dementia (AD) produced fewer verbs in a given time than cognitively healthy older adults (CHOAs; i.e., adults with no history of cognitive decline, cerebrovascular accidents, or other neurological disorders; Alegret et al., 2018; Beber et al., 2015; Davis et al., 2010; Forlenza et al., 2012; Paek et al., 2019) due to the decreased cognitive–linguistic abilities observed in those populations.

Verb fluency can be classified as a type of semantic fluency, because it assesses a participant's ability to generate words related to a specific semantic category, namely, verbs that represent actions that people can perform. However, it differs from other types of semantic fluency, such as noun or category fluency (e.g., animals or supermarket items), because it may involve distinct aspects of language processing, including more complex semantics and syntax. For example, verb fluency tasks may engage processes related to motor aspects of language production, as well as syntactic processes that involve verb-argument structure (Qiu & Johns, 2021; Williams et al., 2021). The constraint in the instructions to produce actions that could be performed by people specifically highlights this motor aspect of language and sets it apart from other forms of semantic fluency. Moreover, verbs generally possess higher semantic complexity compared with nouns, making them a potentially more sensitive measure for detecting semantic declines associated with AD, which could contribute to early detection and better management of the disease (Kim & Thompson, 2004; Williams et al., 2021). Therefore, verb fluency tasks offer important and distinct insights into language abilities in individuals with AD that go beyond what semantic and phonemic fluency measures can capture. Despite its potential benefits, there is currently a dearth of information on verb fluency in AD, in contrast to other types of verbal fluency such as animal fluency or phonemic fluency.

Previous studies indicated that decreased verb fluency is associated with reduced lexical access to verbs and executive dysfunction (Davis et al., 2010; Macoir et al., 2019; Östberg et al., 2005), and thus, the tasks are particularly useful during diagnostic procedures and evaluations of symptoms for people with AD, including the earlier stages of the progressive disorder such as subjective cognitive decline (SCD) and mild cognitive impairment (MCI; Östberg et al., 2005; Zhao et al., 2013). The task is also a useful tool to indicate the progression from SCD to MCI and MCI to AD (Alegret et al., 2018; Östberg et al., 2005) or from Parkinson's disease to Parkinson's disease with dementia (Piatt, Fields, Paolo, Koller & Troster, 1999). Other studies have investigated verb fluency task performance in different types of dementia (e.g., Davis et al., 2010; Delbeuck et al., 2013). Given the increasing number of people diagnosed with Alzheimer's disease and related dementias (Alzheimer's Association, 2022) and the lack of cures for the diseases, there is a critical need for early detection and prevention of the disease and related disorders, as well as intervention and management strategies that are appropriate for the level of cognition in each individual. Therefore, it is imperative to have reliable and valid measures to assist in the evaluation of cognitive and linguistic symptoms, as well as distinguish characteristics of CHOA, SCD, MCI, and AD (Östberg et al., 2005).

There are a variety of methods to analyze verbal fluency performance in addition to calculating the number of correct responses that can further capture cognitive declines. For example, analyses of word clusters (i.e., the size or number of clusters of words that are semantically or phonologically related) may provide additional insights into the cognitive–linguistic characteristics of individuals with Alzheimer's dementia (IwDs). Clusters are consecutive responses grouped together based on predetermined categorization criteria. The average cluster size has been suggested to reflect lexical retrieval skills and semantic networks, whereas the number of switches (i.e., change from one cluster to a different cluster) reflects executive functioning skills such as organization in verbal fluency tasks (Troyer et al., 1997). On the other hand, the number of clusters is thought to reflect both lexical retrieval and executive function abilities (Troyer et al., 1997). Although clustering and switching can drive overall productivity during verbal fluency and are strongly correlated with the total number of correct responses (e.g., Gordon & Chen, 2022), research has demonstrated their potential diagnostic and clinical value in predicting disease progression and differential diagnosis, especially in the context of neurodegenerative disorders such as AD. Previous studies have shown that these measures (switching and clustering) offer unique information about the underlying cognitive processes, highlighting their significance in evaluating individuals with AD (Beatty et al., 1997; Fagundo et al., 2008; Haugrud et al., 2011; Tröster et al., 1998; Weakley & Schmitter-Edgecombe, 2014).

Despite the research analyzing word clusters in verbal fluency tasks, studies investigating clustering and switching have reported a substantial amount of rater bias (Troyer et al., 1997; Vonk et al., 2019). Moreover, verb fluency in particular has less data on cluster analyses and the data available involves methods of clustering that are heterogeneous, making comparison and validation of the method difficult. Furthermore, the lack of research on developing reliable and valid criteria for verb fluency responses lends itself to discrepancies involving issues with interrater and intrarater reliability as well as replication problems. Troyer et al. (1997) and more recent studies (e.g., LeDoux et al., 2014) have developed reliable and valid criteria for the cluster analysis of some types of verbal fluency responses, but it should be noted that these criteria were not specifically designed for verb-based fluency tasks. For example, words with the same first two letters, homophones, rhymes, and so forth are grouped as a cluster in letter fluency tasks (LeDoux et al., 2014). In category fluency, words that belong to the same subcategory or words that obviously hold strong associations in the real world are grouped as a cluster (LeDoux et al., 2014). Category fluency tasks such as animal and supermarket fluency have predetermined categories that are outlined in Troyer et al. (1997), which provide categories for possible correct responses to fit into (e.g., water animals, bovine, African animals). Conversely, there are no predetermined criteria for verbs that have been established, as there are many ways to categorize verbs (e.g., purpose, part of the body that performs the action). Even within these categories, there are no concrete subcategories where a verb will fall into only one category. For example, with no predetermined criteria, the verb “drive” could be categorized as performed by the hands or the feet depending on the rater's opinion. The lack of criteria replicable within or across studies and researchers for verb fluency tasks remains a critical gap in establishing cluster analysis of verb fluency tasks as a reliable and valid diagnostic tool for dementia and other populations.

As mentioned above, previous studies have reported a heterogeneous variety of clustering methods for the verb fluency task. For example, Faroqi-Shah and Milman (2017) grouped responses of 29 individuals with aphasia and 40 neurotypical adults on animal, verb, and letter fluency tasks by using phonemic and semantic relatedness. They defined phonemic relatedness by words that began with the same letter, differed only by a vowel, rhymed, or were homonyms. Semantic relatedness was defined as “a paradigmatically or syntagmatically related response” (Faroqi-Shah & Milman, 2017, p. 374). Although examples specific to verbs were not available from this study, paradigmatically similar words were defined as those from the same subcategory such as dog and cat (pets) or lion and tiger (zoo). Syntagmatically similar words were defined as having a “semantic association such that they often co-occur” (Faroqi-Shah & Milman, 2017, p. 374) such as *salt* and *shaker*. Each participant's responses were inspected and analyzed twice by independent raters who had frequent discussions on how they chose to cluster responses. There was strong interrater reliability (Cohen's *k* = .92), and differences were resolved by a third research assistant.

Macoir et al. (2019) analyzed clusters on the verb fluency and object (noun) fluency responses from 20 older adults with SCD, 20 adults with MCI, and 20 healthy older adults. In this article, the cluster analysis was reported to follow the criteria from Troyer et al. (1997), and the clusters for verbs were determined based on semantic subcategories (e.g., speak, scream, and whisper) and semantic script (e.g., gardening: dig, plant, and rake). There was no specific information provided regarding reliability for verbal fluency tasks.

Another study by McDowd et al. (2011) completed cluster analyses on letter, category, and verb fluency task responses from 36 young adults, 30 healthy older adults, 23 adults with AD, and 30 individuals with Parkinson's disease. For verb fluency, clusters were defined based on subcategories. There were different trials with different prompts for producing verbs such as asking participants to name “things you can do to an egg.” For the “things you can do to an egg” trial, the following categories were used to cluster responses: “ways to cook an egg, ways to destroy an egg, and ways to decorate an egg” (McDowd et al., 2011, p. 11). This was the only criterion described for clustering the verb fluency responses.

The studies mentioned above provide an initial foundation and strong support for the need for replicable criteria for cluster and switch analyses on the verb fluency task. These studies reported heterogeneous criteria that are not easily replicated across studies and appear to rely on subjective methods. Methods used for clustering by Faroqi-Shah and Milman (2017) and Macoir et al. (2019) are similar in that they clustered responses based on semantic features; however, the minimal examples and subjectivity of semantic relatedness makes it difficult to determine whether the two methods will yield similar results. In order to address these difficulties, this study sought to use a predetermined set of norms to examine the feasibility of using objective clustering criteria that can be used to expand upon the work that the previous studies have reported on in order to potentially use the verb fluency task as a reliable evaluation tool. Specifically, we used the Lancaster Sensorimotor Norms (Lynott et al., 2020) as a measure to categorize the verbs. The Lancaster Sensorimotor Norms are a set of semantic norms for English that measure how strongly words are associated with different sensory modalities and action effectors. Thus, the Norms include six perceptual modalities (touch, hearing, smell, taste, vision, and interoception) and five action effectors (mouth/throat, hand/arm, foot/leg, head excluding mouth/throat, and torso). These Norms represent the largest set of semantic norms gathered from 3,500 individual participants for 40,000 words. In Lynott et al. (2020), participants were presented with words and asked to rate their familiarity with the word (e.g., did they know the meaning of the word). Once familiarity was established, participants were asked to categorize the perceptual modalities (“to what extent do you experience WORD”) and action effectors (“to what extend do you experience WORD by performing an action with the…”) for each word when provided with the previously mentioned perceptual modalities and action effectors. High interrater reliability was reported for the perceptual modalities (Cronbach's alpha .90–.96 for each modality) and action effectors (Cronbach's alpha .85–.93 for each effector; Lynott et al., 2020). The dominant dimension was identified as the single highest rating dimension among the dominant perceptual modalities, dominant action effectors, and dominant sensorimotor dimensions (perceptual and action combined).

Using objective norms such as the Lancaster Sensorimotor Norms to cluster verbs has several advantages. It is a large multidimensional data set that can be used to study various aspects of language processing, such as word recognition, categorization, memory, and embodiment (Lynott et al., 2020). Additionally, it allows for quantifying the strength of verb–action effector associations, which are important dimensions of verb meaning. Using the Lancaster Norms in clustering verbs also has the advantage of being more reliable and consistent than subjective methods that rely on human ratings, which may vary depending on individual experience and subjective judgment. Moreover, the embodied cognition framework suggests that language is shaped by our bodily experiences and interactions with the world (Gallese & Cuccio, 2018). Thus, using sensorimotor norms to cluster verbs may shed further insights into the cognitive–linguistic strategies that people use when generating verbs in a fluency task.

In a recent study by Qiu and Johns (2021), the Lancaster Norms were used as an objective measure to determine sensorimotor similarity of responses from healthy adults on verbal fluency tasks including verb and noun fluency. They found that the produced verbs were more widely distributed across multiple modalities and sensorimotor effectors (i.e., different sensory and motor systems, which include vision, hearing, smell, taste, touch, and various motor actions) compared with the nouns generated during the fluency tasks. This indicates a broader array of sensory experiences and motor actions associated with the generated verbs. Conversely, the nouns produced demonstrated more homogeneity, suggesting that they were more likely to share similar sensory experiences and actions. Thus, noun and verb fluencies reflect different aspects of lexical knowledge and retrieval processes, and using sensorimotor norms can provide valuable insights into how verbs are stored and retrieved during verb fluency.

By using the Lancaster Norms, which measure how much words evoke different sensations and actions, we will be able to explore how to cluster verbs based on their sensorimotor features. However, there are also potential limitations to using these Norms. For example, they may not capture all the nuances and variability of sensorimotor experiences across individuals and contexts. Additionally, although the Lancaster Norms can capture some aspects of word associations based on sensorimotor experiences, they might not capture other aspects like phonological similarities. Nevertheless, given the lack of objective criteria and understanding related to different approaches to measuring verb fluency performance, this study used the Lancaster Norms as the objective clustering method considering the large participant sample and word bank, and high reliability with its potential contribution to the development of more effective methods.

In addition to needing predetermined clustering criteria to increase the reliability and validity of verb fluency tasks, the previous literature highlights the need for well-established interrater and intrarater reliability within the studies. Faroqi-Shah and Milman (2017) reported very strong interrater reliability (Cohen's *k* = .92) on all verbal fluency tasks collapsed across animals, verb, and letter categories. This indicates that raters who participated in the clustering method had a very strong agreement in the clusters on a randomly selected 20% of the sample. There was no intrarater reliability reported in either of the other two studies highlighted in this article for clustering analyses on the verb fluency task. For example, no specific information for the action category was provided by McDowd et al. (2011), whereas they provided that Cronbach's alpha was greater than .90 for clusters, switches, and cluster size of all fluency tasks including letter, semantic, and action fluency tasks.

Therefore, this study also aimed to compare (a) the interrater reliability between subjective analyses of the two raters (first and last author) to determine the reliability of traditional methods and (b) the intermethod reliability between subjective and objective analyses to measure the degree to which these two methods converge. The subjective clustering methods used in this article were an attempt to replicate the methods used by Macoir et al. (2019), McDowd et al. (2011), and Faroqi-Shah and Milman (2017) by instructing raters to cluster words with semantic relationships. The objective clustering method utilized the Lancaster Norms (Lynott et al., 2020) as a predetermined categorization of verbs that removes a potential for rater bias (Vonk et al., 2019) or inconsistency within and across raters. This study also investigated intrarater reliability in human raters to further establish reliability within the subjective method before comparing it with the objective method.

Based on the current literature, we hypothesized that the objective clustering method would reveal a significant difference in the performance on the verb fluency task between IwDs and CHOAs for the number of clusters, average cluster size, and number of switches in that semantic impairments are commonly observed in IwDs (Balthazar et al., 2008; Bayles et al., 1990; Henry et al., 2004). We also hypothesized that the interrater reliability between two raters using the subjective clustering methods would be weak given the ambiguous clustering instructions and possible rater bias. Similarly, we hypothesized that the intermethod reliability for the objective and subjective clustering methods would also be weak given the differences in clustering methods.

## Method

Twenty IwDs and 22 CHOAs participated in this study. Participants were recruited from a large, community-based study of aging and neurodegenerative diseases. The inclusionary criteria for participants with dementia included a clinical diagnosis of amnestic phenotype of AD meeting the criteria of McKhann et al. (2011) based on results of neuropsychological and neurological assessments, mild or moderate severity (mild > 21; 11 < moderate < 20; Perneczky et al., 2006) demonstrated by MMSE-2 scores (Folstein et al., 2010), and no history of other neurological or psychiatric diseases that might affect communication abilities (e.g., schizophrenia, stroke-induced aphasia, traumatic brain injury, epilepsy, and clinical depression) as well as no history of alcohol or drug abuse. Additionally, the inclusionary criteria for the CHOA group included no history of neurological, psychiatric, or developmental disorders (e.g., schizophrenia, stroke, traumatic brain injury, epilepsy, and clinical depression) or substance abuse that may result in communication disorders. All participants were native speakers of English and right-handed, with normal hearing and vision (corrected or uncorrected). The Speech Discrimination Screening subtest from Arizona Battery for Communication Disorders of Dementia (ABCD; Bayles & Tomoeda, 1993) was used for hearing screening. Vision was screened through a picture-matching task. This study was approved by the institutional review board at the University of Tennessee Health Science Center and part of a larger research project.

Table 1 shows demographic information for the two groups including age, gender, years of education, and MMSE scores. The education level did not differ between the two groups (*p* = .971). Also, the gender distribution between the two groups did not differ (*p* = .43). As expected, the MMSE-2 scores were significantly lower in the dementia group compared with the CHOA group (*p <* .001). A statistically significant group difference was also discovered for age (*p* = .015). To ensure the reliability of our analysis, we conducted secondary analyses on a subset of data, which excluded three outliers (two CHOAs aged 64 years and one IwD aged 94 years). With this secondary data set with no group difference in age (*t* = −1.88, *df* = 37, and *p* = .069), the results for all analyses remained consistent (i.e., *t* tests and intraclass correlation coefficients [ICCs]). The findings of these supplementary analyses enhance the reliability of our statistical results, thus substantiating that the observed distinctions between the control and the AD groups can be attributed to cognitive impairments. All participants were instructed to do the following for the verb fluency task, as first introduced by Piatt, Fields, Paolo, and Troster (1999):

**Table 1.** Demographic information on the individuals with dementia (IwDs) and cognitively healthy older adults (CHOAs).

Variable	IwDs (*n* = 20)	CHOA (*n* = 22)	Group difference	*p*
Age	78.15 (8.02)	72.27 (6.94)	*t*(40) *=* −2.55*	.015
Education	18.13 (13.41)	18.27 (12.43)	*t*(40) *=* 0.04	.971
MMSE-2	22.79 (4.79)	27.73 (1.49)	*t*(20.99) *=* 4.32*^a^	< .001
Gender	F = 15, M = 5	F = 14, M = 8	χ^2^ (1, *Ν* = 42) = .63	.43

*Note.* MMSE-2 = Mini-Mental State Examination-2; F = female; M = male.

a Equal variances not assumed.

* *p* < .01.

*I'd like you to tell me as many different things as you can think of that people do. I don't want you to use the same word with different endings, like eat, eating, eaten. Also, just give me single words such as eat, or smell, rather than a sentence. Can you give me an example of something that people do?*

If the response was unacceptable, participants were asked to provide a different example. If the response was an acceptable verb, participants were further instructed:

*That's the idea. Now in 30 seconds, tell me as many different things as you can think of that people do.*

Responses were then transcribed verbatim via video and audio recording of the responses to ensure correct transcription, and interrater reliability for transcription and scoring on 20% of the sample was 100%.

From the recorded responses, only correct responses from this task were used in all analyses to investigate the lexical retrieval and cognitive processes used strictly for verbs. Incorrect responses (i.e., words belonging to a different grammatical class), perseverations, or extraneous comments were removed before analyses were completed. Two types of clustering methods, objective and subjective, were utilized to analyze the data in this study. Both methods used a task congruent method to cluster responses. A task congruent method of clustering includes clustering either semantically related words for a category fluency task (i.e., verb fluency or animal fluency) or phoneme-relatedness criteria for letter fluency tasks (Lehtinen et al., 2021). The objective clustering method utilized the Lancaster Norms database (Lynott et al., 2020) to analyze clusters and switches. The Lancaster Norms classify over 40,000 English verbs into one of five categories of action effectors: hand/arm, head excluding mouth/throat, foot/leg, mouth, and torso. Based on the Lancaster Norms, one dominant action effector was distinguished as having the highest rating within the action effectors. Then, the two groups (AD and CHOAs) were compared using independent-samples *t* tests for the following variables: total number of words (correct responses), number of clusters, average cluster size, and number of switches.

The number of clusters was calculated by counting the total number of clusters (consecutive responses in the same category) for each individual participant. The average cluster size for each participant was calculated by dividing the sum of cluster sizes for individual participants by the total number of clusters for that participant. Cluster sizes were calculated by counting correct responses starting at the second word in the cluster, so there were no clusters with a cluster size of zero. That is, a cluster size was determined by subtracting 1 from the total number of words in each cluster. For example, the cluster size of a cluster consisting of two words *run* and *jump* would be coded as 1. The number of switches was calculated by counting the number of shifts between each action effector from the Lancaster Norms (or in the case of subjective methods, different parts of the body that perform the verb), which would include responses that did not have consecutive congruent action effectors (or in the subjective rating, congruent parts of the body that completed the action). That is, single word responses not belonging to any clusters were counted in calculating switches to capture transitions between each word or cluster (Macoir et al., 2019; McDowd et al., 2011; Troyer et al., 1997).

The subjective clustering method utilized two raters categorizing the responses for each group (IwD and CHOA) based on the previous studies (Faroqi-Shah & Milman, 2017; Macoir et al., 2019; McDowd et al., 2011). Both raters analyzed the responses for each participant using the following instructions on an Excel spreadsheet.

A cluster is defined as a sequential group of words with a commonality (semantics). Based on this definition of a cluster, you will be clustering (grouping) responses on a verb fluency task where participants were asked to name as many things as possible that “people do” (verbs) within a time frame of 30 s. Start with the first response and mark it in Cluster 1. Then move to the next response and determine if there is a relationship between the two words. If there is, put it in Cluster 1 and write a word or short phrase that describes the commonality. If there is not a commonality, move on to the following response and assess its commonality with the response that appears next. Repeat this for all of the responses. There is no limit on how small or large the cluster can be.

An example for an animal fluency task was provided to avoid priming any categories for verbs and to capture the raters' initial categorization methods more naturally. Raters were reminded to complete this independently and to be as consistent as possible between each participant. To analyze the interrater reliability between the two raters, an addition was made to the above instructions, which is to categorize the verbs based on the part of the body used to complete the verb. The entire data set was analyzed by the first and third authors. In order to establish intrarater reliability, the first author completed the clustering analysis twice, 2 weeks apart. ICCs (Shrout & Fleiss, 1979) using a single-measurement, absolute-agreement, two-way random-effects model were calculated for (a) intrarater reliability (first author's); (b) interrater reliability (between first and third authors) for subjective methods; (c) intermethod reliability (between first author's subjective method and objective method); and (d) intermethod reliability (between third author's subjective method and objective method) on the number of clusters, average cluster size, and number of switches. The level of reliability was interpreted according to Koo and Li (2016) benchmarks: poor (less than .5), moderate (.5–.75), good (.75–.90), and excellent (.90). Bivariate correlational analyses were also conducted to show the relationships between our measures (see Supplemental Materials S1–S3). All analyses were conducted using SPSS 27 software.

## Results

The responses for both the AD and CHOA groups were first analyzed using the objective clustering method (i.e., using Lancaster Norms), and the means were compared using independent-samples *t* tests on the following variables: number of clusters, average cluster size, number of switches, and total number of words (see Table 2). Compared with CHOAs, IwDs produced significantly fewer words (CHOA *M* = 12.27, *SD =* 1.89; IwD *M* = 7.65, *SD* = 3.28; *t*[40] = 5.66, *p* < .001), significantly fewer clusters (CHOA *M =* 2.64, *SD =* 1.14*;* IwD *M* = 1.60, *SD =* 1.10; *t*[40] = 3.00, *p* = .005), and significantly fewer switches (CHOA *M =* 6.50, *SD* = 1.63; IwD *M* = 4.10, *SD =* 2.13; *t*[40] = 4.13, *p* < .001). There was no significant difference between the CHOAs (*M* = 1.90, *SD =* .77) and IwDs (*M =* 1.41, *SD =* 1.14) regarding cluster size, *t*(40) = 1.65, *p* = .108. These findings were mostly consistent with those obtained from human raters who used the subjective clustering method (see Table 2 and Supplemental Material S4).

**Table 2.** Group comparison of verb fluency responses using the objective and subjective clustering methods.

Variable	IwDs	CHOA	*t* (*df* = 40)	*p*
Total number of words	7.65 (3.28)	12.27(1.89)	5.53*^a^	< .001
Number of clusters				
Objective (LN)	1.60 (1.10)	2.64 (1.14)	3.00*	.005
Subjective (Rater 1)	1.50 (1.05)	2.91 (1.27)	3.90*	< .001
Subjective (Rater 2)	1.60 (.99)	3.09 (1.34)	4.06*	< .001
Cluster size				
Objective (LN)	1.41 (1.14)	1.90 (0.77)	1.65	.108
Subjective (Rater 1)	1.51 (1.10)	1.77 (1.04)	0.78	.440
Subjective (Rater 2)	1.33 (1.83)	1.96 (0.77)	2.56*	.014
Number of switches				
Objective (LN)	4.10 (2.13)	6.50 (1.63)	4.13*	< .001
Subjective (Rater 1)	4.20 (2.19)	6.41 (2.11)	3.33*	.002
Subjective (Rater 2)	4.20 (1.74)	5.45 (1.38)	2.61*	.013

*Note.* IwDs = individuals with Alzheimer's dementia; CHOAs = cognitively healthy older adults; LN = Lancaster Norms.

a Equal variances not assumed (*df* = 29.66).

* *p* < .05.

Additionally, all ICCs for intrarater reliability resulted in moderate to excellent correlations (see Table 3), indicating strong reliability within the first author when subjectively categorizing verb fluency responses. For the number of clusters, the ICC for intrarater reliability was .92, with the confidence interval (the level of confidence for all intervals was 95%) ranging from moderate to excellent [.68, .98]. The ICC for interrater reliability (number of clusters) between the two authors was .81, with the confidence interval ranging from moderate to good [.68, .89] (Koo & Li, 2016). The ICC for intermethod reliability (number of clusters) between the objective and subjective clustering methods was .80 (Rater 1 and objective) and .77 (Rater 2 and objective), with the confidence interval ranging from moderate to good on both ([.69, .89] for Rater 1; [.61, .89] for Rater 2). For the number of switches, the intrarater reliability was .93, with the confidence interval ranging from moderate to excellent [.69, .99]. The ICC for interrater reliability (number of switches) between the two authors was .69, with the confidence interval ranging from poor to good [.49, .82]. The ICC for intermethod reliability (number of switches) between the objective and subjective clustering methods was .76 (Rater 1 and objective) and .70 (Rater 2 and objective) with the confidence intervals ranging from moderate to good ([.60, .87] for Rater 1; [.50, .83] for Rater 2). For the average cluster size, the ICC for intrarater reliability was .98, with the confidence interval ranging from good to excellent [.89, .99]. The ICC for the interrater reliability (average cluster size) between the two authors was .51, with the confidence interval ranging from poor to moderate [.24, .70]. The ICC for the intermethod reliability (average cluster size) between the objective and subjective clustering methods was .64 (Rater 1 and objective) and .53 (Rater 2 and objective), with confidence intervals ranging from poor to good ([.42, .79] for Rater 1; [.27, .72] for Rater 2).

**Table 3.** Intraclass correlation coefficients for the variables on verb fluency.

Variables		ICC	95% CI
Number of clusters	Intrarater (within Rater 1)	.92*	[.68, .98]
	Interrater (between Raters 1 and 2)	.81*	[.68, .89]
	Intermethod (between objective and Rater 1)	.80*	[.69, .89]
	Intermethod (between objective and Rater 2)	.77*	[.61, .89]
Average cluster size	Intrarater (within Rater 1)	.98*	[.89, .99]
	Interrater (between Raters 1 and 2)	.51*	[.24, .70]
	Intermethod (between objective and Rater 1)	.64*	[.42, .79]
	Intermethod (between objective and Rater 2)	.53*	[.27, .72]
Number of switches	Intrarater (within Rater 1)	.93*	[.69, .99]
	Interrater (between Raters 1 and 2)	.69*	[.49, .82]
	Intermethod (between objective and Rater 1)	.76*	[.60, .87]
	Intermethod (between objective and Rater 2)	.70*	[.50, .83]

*Note.* ICC *=* intraclass correlation coefficient; CI = confidence level.

* *p* < .005. According to Koo and Li (2016), the level of reliability was determined with the following criteria: below .5 = poor, between .50 and .75 = moderate, between .75 and .90 = good, and above .90 = excellent.

## Discussion

We investigated whether objective clustering methods can be used to compare verb fluency performance in IwDs and CHOAs and examined the intrarater, interrater, and intermethod reliability within and across raters and for different clustering methods. Using the objective clustering method, the IwDs produced significantly fewer clusters and switches. Although the IwDs produced smaller clusters on average, there was no significant difference with the CHOA group. The subjective clustering method yielded strong intrarater reliability and variable interrater and intermethod reliability between the authors as well as between the objective and subjective clustering methods.

Looking at the interrater and intrarater reliability analyzed in this study, subjective clustering methods produced excellent intrarater reliability on all variables. This indicates that one rater can repeatedly categorize the responses similarly on different occasions—an important consideration for assessment particularly when tracking disease progression or treatment effectiveness over time. The subjective clustering methods revealed questionable interrater reliability, especially on the average cluster size variable in comparison with other variables. Such potential for inconsistency is problematic for a reliable and valid assessment. Previous studies that have investigated the interrater reliability between two raters for clustering/switching analyses for other verbal fluency tasks (i.e., animal fluency) reported strong correlations overall (LeDoux et al., 2014; Ross et al., 2007). These studies that have reported interrater reliability for clustering and switching analyses on animal fluency or letter fluency tasks have utilized criteria from Troyer et al. (1997) and LeDoux et al. (2014), establishing widely accepted and replicable criteria, which could contribute to the higher interrater reliability observed in those cluster analyses. Given the variability of the interrater reliability between two raters reported in this study during a verb fluency task, there appears to be increased opportunities for rater inconsistency especially when the clustering criteria are vague.

Additionally, the ICCs between the subjective and objective clustering methods were classified as moderate (for the cluster size) or good (for the number of clusters and switches) according to Koo and Li (2016). This indicates that there might be a commonality between the two clustering methods lending some support to the validity of the objective clustering method, but not strong enough ICC to claim that both methods would yield the same results. This also suggests that the two methods may capture different aspects of verb semantics compared with human raters. Upon comparison, we observed that the disparities between the two methods can be ascribed to the more elaborate categorization used by either the objective or subjective clustering approach (although these differences do not encompass all possible explanations for the observed disparities; thus, further research is warranted).

For example, although human raters would cluster *talk*, *laugh*, and *cry* together as a cluster associated with face, the Lancaster Norms used a more detailed categorization, with *talk* and *laugh* clustered for mouth and *cry* for head (see Table 4). On the other hand, *garden*, *swim*, and *plant* were all clustered together by the Lancaster Norms into the category of hand (arm), whereas human raters agreed that *swim* belongs to the whole body, and the other two (*garden* and *plant*) belong either to arms or hands. However, the relatively weak concordance between objective and subjective methods is comparable with the degree of reliability observed between two human raters (i.e., ICCs between the human raters). That is, the discrepancies between objective and subjective methods are not unique to the comparison between objective and subjective methods. This underscores the need for further research on a comprehensive and reliable set of criteria for cluster analysis and how sensorimotor features can contribute to verb processing and semantics in establishing the criteria. Because the use of Lancaster Norms contributed to differentiating the two groups in terms of the number of clusters and switches, which is consistent with subjective clustering methods, this method may be another viable option in clinical diagnostic processes to investigate cognitive processes underlying verbal fluency impairment.

**Table 4.** Example of participant responses and clustering and switch analyses.

Variables	Example from a CHOA					Example from a participant with AD				
	Response	Score	Lancaster Norms^a^	Rater 1	Rater 2	Response	Score	Lancaster Norms^a^	Rater 1	Rater 2
	Talk	1	***mouth***	***face***	***mouth***	Garden	1	***hand_arm***	arms	hands
	Smile	1	***mouth***	***face***	***mouth***	Swim	1	***hand_arm***	whole body	whole body
	Cry	1	head	***face***	***mouth***	Plant	1	***hand_arm***	arms	hands
	Laugh	1	mouth	***face***	***mouth***	Walk	1	foot_leg	legs	legs
	Run	1	***foot_leg***	***legs***	***legs***	Talk	1	***mouth***	***face***	***face***
	Jump	1	***foot_leg***	***legs***	***legs***	Laugh	1	***mouth***	***face***	***face***
	Walk	1	***foot_leg***	***legs***	***legs***	Cry	1	head	***face***	***face***
	Talk^b^	0	n/a	n/a	n/a					
	Blink	1	head	***face***	face					
	Swallow	1	mouth	***face***	***mouth***					
	Sneeze	1	head	***face***	***mouth***					
	Clap	1	hand_arm	arms	***hand***					
	Count	1	head	mind	***hand***					
	Swim	1	hand_arm	whole body	whole body					
Number of clusters			2	3	4			2	1	1
Average cluster size			1.5	2.33	1.75			1.5	2	2
Number of switches			9	5	5			3	4	4

*Note.* Responses in the italicized areas indicate that they were clustered together. The bold text symbolizes clusters per each rater. CHOA *=* cognitively healthy older adult; AD = Alzheimer's dementia.

a The dominant action effector was determined using the Lancaster Norms database.

b This is an incorrect response and therefore was not included in the analysis.

Although clustering and switching analyses from category fluency tasks have been extensively studied in neurological populations, verb clustering and switching analyses have received limited attention in research. Because these measures provide valuable insight into cognitive processes underlying impaired verbal fluency performance, exploring verb fluency abilities through cluster and switch analyses can significantly enhance our understanding of AD beyond traditional accuracy-based scoring approach. Similar to the findings of Macoir et al. (2019), this study reports no significant difference between people with neurodegenerative disorders and CHOAs regarding the average cluster size in verb fluency responses. With other verbal fluency tasks such as animal fluency, there are variable results regarding the group differences with respect to average cluster size in IwDs compared with CHOAs. Some studies have indicated that IwDs (or those with MCI) do not produce significantly smaller cluster sizes in comparison with healthy controls (e.g., Peter et al., 2016; Raoux et al., 2008; Tröger et al., 2019; Weakley et al., 2013). On the other hand, other studies have reported a significant difference between the two groups regarding average cluster size with IwDs producing significantly smaller cluster sizes (e.g., Murphy et al., 2006; Oh et al., 2019). Tröger et al. (2019) reported a lack of statistically significant differences on the average cluster size during the animal fluency task although CHOAs produced the largest cluster sizes, followed by MCI participants, and IwDs produced the smallest cluster sizes (i.e., AD < MCI < CHOA). They concluded that these results could highlight semantic memory retrieval processes in IwDs being inefficient, but still effective, which could be present in this study considering similar results on a different verbal fluency task.

Another possible explanation for the lack of significance in group differences for the average cluster size is the heterogeneous presentation of AD. The large standards of deviation in the IwDs, especially for the total number of words (*SD* = 3.28) and number of switches (*SD* = 2.13) reflect the heterogeneity of verb fluency performance in the dementia population even within the mild and moderate stages of the disorder and precipitating factors, which has been reported in previous studies (Delbeuck et al., 2013). As the heterogeneity of AD is commonly observed and has been reported previously, the objective clustering method used is a viable option despite the lack of group differences on the average cluster size variable.

With the objective clustering method, there were significantly fewer switches in IwDs compared with CHOAs. These results are consistent with previous category fluency studies using animal fluency (Raoux et al., 2008; Rofes et al., 2020; Tröger et al., 2019), suggesting that objective clustering methods focusing on action semantics are applicable to analyses of verb fluency responses. For example, Tröger et al. (2019) reported a significant difference between groups on an animal fluency task where IwDs switched significantly less than MCI and CHOA groups (IwDs < MCI = CHOA), highlighting the different cognitive characteristics of CHOAs and IwDs. Switching is considered to be a more cognitively demanding skill than clustering, as it involves “higher cognitive functions, such as cognitive flexibility and strategic search processes” (Lehtinen et al., 2021, p. 3). Troyer et al. (1998) reported that participants with frontal lobe lesions demonstrated less switching than healthy controls, indicating that executive functioning difficulties could be a factor in the decreased performance on verbal fluency tasks. Paek et al. (2020) reported that verb retrieval involves activation in parts of the brain including the hippocampus and frontal lobes with the frontal lobe typically having associations with executive function abilities. In the early stages of AD, there is typically damage to the temporal regions of the brain extending to the parietal and frontal lobes and ultimately leading to global neurodegeneration (Koenig et al., 2018). Since neuropathological changes to the frontal lobe are commonly observed in IwDs, there could be a decrease in the executive function skills that are used during the verb fluency task observed in this population. These executive function skills such as switching, inhibition, and organizing may be reflected in the number of clusters and number of switches variables. However, it should be noted that the extent to which clustering and switching provide information about AD over and above the total number of items produced is currently unclear. Some studies have claimed that these measures offer unique predictive value (Raoux et al., 2008; Tröster et al., 1998), although others have found that they do not significantly improve prediction once total output is taken into account (Gordon & Chen, 2022).

## Limitations and Future Research

Although this study investigated the use of the Lancaster Norms as an objective method for clustering verbs in a verb fluency task, there are other potential methods for verb clustering that may also prove to be reliable. An additional or novel method that could be utilized for clustering the verb fluency task includes why someone performs the verb (e.g., work, leisure, hygiene). Although task congruent clustering can provide insight into the categorization and organization during generative naming, it is possible that other clustering methods (i.e., task discrepant clusters) could be used as a strategy during analyses of verbal fluency responses. An example of a task-discrepant cluster for a category fluency task would be using a phonemic relatedness criterion for clustering methods (e.g., words that start with the same letter and then switching to another letter) or using a semantically relatedness criterion for a letter fluency task (e.g., words that belong to the same subcategory), which was reported as part of Faroqi-Shah and Milman (2017)'s criteria (Lehtinen et al., 2021). By choosing to use a task-congruent method, the results of this study can be reported in the context of the clustering method used but cannot be generalized to all clustering methods as they were not analyzed. To effectively evaluate clustering in the verb fluency task, it may be beneficial to compare different clustering criterion with task-congruent and task-discrepant methods in future studies to better capture the cognitive processes utilized by participants during the verb fluency task. Using both task-congruent and task-discrepant methods of clustering methods would impact the number of clusters, average cluster size, number of switches, and interrater and intrarater reliability for both IwDs and CHOAs. A combination of different clustering criteria for the verb fluency responses could provide insight on the connectedness of different methods and the methods that most accurately highlight the lexical retrieval and executive function processes used by participants. Additionally, the instructions used in an attempt to replicate Piatt, Fields, Paolo, and Troster's (1999) original instructions limit the responses to the verb fluency task to be actions that can be done by people. This could limit the responses to not include actions that can be done by other agents.

Another future direction that could highlight group differences between IwDs and CHOAs is using temporal analyses to determine whether there are any patterns or differences in response time (e.g., initial response time, interword response time, using intervals to analyze how many words were produced in specific intervals). This method could provide more insight into the executive functioning deficits, which has been investigated in other studies where IwDs tend to take a longer time to produce words in animal and letter fluency (Tröger et al., 2019). The temporal analysis could be used in conjunction with clustering and switching analyses to investigate any relationships between temporal results and clustering and switching variables during the verb fluency task. Additionally, in order to gain deeper insights into the source of discrepancies between the two methods (as well as among human raters), it would be beneficial to conduct item-based and qualitative analyses.

Other limitations include a small sample size, as a larger sample size would strengthen the results and make them more generalizable to the participant population. It would also be beneficial for the verb fluency clustering analyses to be used with other populations that experience neuropathological changes such as Parkinson's disease, primary progressive aphasia, and other types of dementia with a standardized method for clustering.

Overall, the verb fluency task continues to provide insight into lexical retrieval and executive function abilities in IwDs and CHOAs; however, there is a need for a stricter protocol when clustering responses to the verb fluency task to eliminate the subjectivity that hinders replication across studies. Subjective clustering methods should be used with caution and researchers will need to consider methodological validity in cluster analyses and/or continue to investigate objective clustering methods, with the Lancaster Norms being a viable and replicable method for cluster analyses in future studies.

## Data Availability Statement

The data sets generated during and/or analyzed during the current study are available from the corresponding author on reasonable request.

## Supplementary Material

10.1044/2023_AJSLP-22-00290SMS1 Supplemental Material S1 Correlations between all variables across all participants.

10.1044/2023_AJSLP-22-00290SMS2 Supplemental Material S2 Correlations between all variables in cognitively healthy older adults.

10.1044/2023_AJSLP-22-00290SMS3 Supplemental Material S3 Correlations between all variables in participants with Alzheimer's Disease.

10.1044/2023_AJSLP-22-00290SMS4 Supplemental Material S4 Detailed data.
