# Supplementary material for: Objective and Subjective Clustering Methods for Verb Fluency Responses From Individuals With Alzheimer's Dementia and Cognitively Healthy Older Adults
Source: Am J Speech Lang Pathol. 2023 Sep 18;32(5 Suppl):2589–601. doi: 10.1044/2023_AJSLP-22-00290 (PMC10721246; doi:10.1044/2023_AJSLP-22-00290)
Supplement: Supplemental Material S1 [file AJSLP-32-2589-s001.pdf]

Supplemental material, Fisher et al., "Objective and subjective clustering methods for verb fluency responses from individuals with Alzheimer's dementia and cognitively healthy older adults," *AJSLP*, https://doi.org/10.1044/2023_AJSLP-22-00290

Supplemental Material S1. Correlations between all variables across all participants.

		age	education	MMSE	#correct	#cluster_H1	#cluster_LN	#cluster_H2	cluster size_H1	cluster size_LN	cluster size_H2	#switch_H1	#switch_LN	#switch_H2	%cluster_H1	%cluster_LN	%cluster_H2	%switch_H1	%switch_LN	%switch_H2
age	Pearson Correlation	1	–.082	–.321*	–.124	–.129	–.081	–.141	.065	.035	–.009	–.056	–.012	.036	–.026	.154	.008	.032	–.003	.119
	Sig. (2-tailed)		.619	.046	.452	.435	.622	.392	.696	.834	.954	.737	.942	.828	.877	.349	.963	.848	.983	.469
	N	39	39	39	39	39	39	39	39	39	39	39	39	39	39	39	39	39	39	39
education	Pearson Correlation	–.082	1	–.131	–.080	.047	.072	.054	–.207	–.225	–.281	–.079	–.119	–.140	–.088	–.085	–.073	.050	.039	.019
	Sig. (2-tailed)	.619		.425	.629	.774	.662	.742	.206	.168	.084	.631	.470	.394	.595	.608	.657	.763	.812	.906
	N	39	39	39	39	39	39	39	39	39	39	39	39	39	39	39	39	39	39	39
MMSE	Pearson Correlation	–.321*	–.131	1	.608**	.435**	.462**	.407*	.272	.234	.467**	.397*	.463**	.385*	.283	.251	.254	–.111	–.070	–.393*
	Sig. (2-tailed)	.046	.425		.000	.006	.003	.010	.094	.152	.003	.012	.003	.016	.081	.124	.119	.502	.672	.013
	N	39	39	39	39	39	39	39	39	39	39	39	39	39	39	39	39	39	39	39
#correct	Pearson Correlation	–.124	–.080	.608**	1	.787**	.746**	.825**	.231	.412**	.495**	.684**	.793**	.672**	.433**	.389*	.582**	–.031	–.075	–.593**
	Sig. (2-tailed)	.452	.629	.000		.000	.000	.000	.156	.009	.001	.000	.000	.000	.006	.014	.000	.852	.650	.000
	N	39	39	39	39	39	39	39	39	39	39	39	39	39	39	39	39	39	39	39
#cluster_H1	Pearson Correlation	–.129	.047	.435**	.787**	1	.803**	.829**	.038	.230	.266	.388*	.533**	.456**	.826**	.496**	.647**	–.247	–.166	–.520**
	Sig. (2-tailed)	.435	.774	.006	.000		.000	.000	.819	.160	.102	.015	.000	.004	.000	.001	.000	.130	.313	.001
	N	39	39	39	39	39	39	39	39	39	39	39	39	39	39	39	39	39	39	39
#cluster_LN	Pearson Correlation	–.081	.072	.462**	.746**	.803**	1	.780**	.088	.136	.298	.485**	.400*	.422**	.564**	.820**	.634**	–.063	–.336*	–.529**
	Sig. (2-tailed)	.622	.662	.003	.000	.000		.000	.596	.410	.065	.002	.012	.008	.000	.000	.000	.702	.037	.001
	N	39	39	39	39	39	39	39	39	39	39	39	39	39	39	39	39	39	39	39
#cluster_H2	Pearson Correlation	–.141	.054	.407*	.825**	.829**	.780**	1	.023	.265	.133	.589**	.607**	.500**	.558**	.476**	.872**	–.009	–.119	–.523**
	Sig. (2-tailed)	.392	.742	.010	.000	.000	.000		.888	.102	.419	.000	.000	.001	.000	.002	.000	.955	.471	.001
	N	39	39	39	39	39	39	39	39	39	39	39	39	39	39	39	39	39	39	39
cluster size_H1	Pearson Correlation	.065	–.207	.272	.231	.038	.088	.023	1	.686**	.523**	–.328*	–.099	–.111	.080	.121	–.007	–.729**	–.609**	–.439**
	Sig. (2-tailed)	.696	.206	.094	.156	.819	.596	.888		.000	.001	.041	.549	.503	.628	.464	.968	.000	.000	.005
	N	39	39	39	39	39	39	39	39	39	39	39	39	39	39	39	39	39	39	39
cluster size_LN	Pearson Correlation	.035	–.225	.234	.412**	.230	.136	.265	.686**	1	.522**	.029	.014	.124	.161	.132	.279	–.398*	–.662**	–.474**
	Sig. (2-tailed)	.834	.168	.152	.009	.160	.410	.102	.000		.001	.861	.930	.453	.327	.424	.085	.012	.000	.002
	N	39	39	39	39	39	39	39	39	39	39	39	39	39	39	39	39	39	39	39
cluster size_H2	Pearson Correlation	–.009	–.281	.467**	.495**	.266	.298	.133	.523**	.522**	1	.091	.217	.017	.138	.256	.056	–.242	–.312	–.725**
	Sig. (2-tailed)	.954	.084	.003	.001	.102	.065	.419	.001	.001		.582	.184	.918	.402	.116	.735	.138	.053	.000
	N	39	39	39	39	39	39	39	39	39	39	39	39	39	39	39	39	39	39	39
#switch_H1	Pearson Correlation	–.056	–.079	.397*	.684**	.388*	.485**	.589**	–.328*	.029	.091	1	.781**	.814**	.070	.255	.461**	.627**	.305	–.060
	Sig. (2-tailed)	.737	.631	.012	.000	.015	.002	.000	.041	.861	.582		.000	.000	.673	.117	.003	.000	.059	.718
	N	39	39	39	39	39	39	39	39	39	39	39	39	39	39	39	39	39	39	39
#switch_LN	Pearson Correlation	–.012	–.119	.463**	.793**	.533**	.400*	.607**	–.099	.014	.217	.781**	1	.778**	.236	.064	.405*	.285	.500**	–.201
	Sig. (2-tailed)	.942	.470	.003	.000	.000	.012	.000	.549	.930	.184	.000		.000	.149	.701	.011	.079	.001	.221
	N	39	39	39	39	39	39	39	39	39	39	39	39	39	39	39	39	39	39	39
#switch_H2	Pearson Correlation	.036	–.140	.385*	.672**	.456**	.422**	.500**	–.111	.124	.017	.814**	.778**	1	.228	.212	.373*	.355*	.252	.176
	Sig. (2-tailed)	.828	.394	.016	.000	.004	.008	.001	.503	.453	.918	.000	.000		.162	.196	.019	.027	.122	.284
	N	39	39	39	39	39	39	39	39	39	39	39	39	39	39	39	39	39	39	39
%cluster_H1	Pearson Correlation	–.026	–.088	.283	.433**	.826**	.564**	.558**	.080	.161	.138	.070	.236	.228	1	.456**	.543**	–.467**	–.204	–.325*
	Sig. (2-tailed)	.877	.595	.081	.006	.000	.000	.000	.628	.327	.402	.673	.149	.162		.004	.000	.003	.213	.044
	N	39	39	39	39	39	39	39	39	39	39	39	39	39	39	39	39	39	39	39
%cluster_LN	Pearson Correlation	.154	–.085	.251	.389*	.496**	.820**	.476**	.121	.132	.256	.255	.064	.212	.456**	1	.563**	–.022	–.500**	–.343*
	Sig. (2-tailed)	.349	.608	.124	.014	.001	.000	.002	.464	.424	.116	.117	.701	.196	.004		.000	.894	.001	.032
	N	39	39	39	39	39	39	39	39	39	39	39	39	39	39	39	39	39	39	39
%cluster_H2	Pearson Correlation	.008	–.073	.254	.582**	.647**	.634**	.872**	–.007	.279	.056	.461**	.405*	.373*	.543**	.563**	1	.061	–.180	–.387*
	Sig. (2-tailed)	.963	.657	.119	.000	.000	.000	.000	.968	.085	.735	.003	.011	.019	.000	.000		.712	.273	.015
	N	39	39	39	39	39	39	39	39	39	39	39	39	39	39	39	39	39	39	39
%switch_H1	Pearson Correlation	.032	.050	–.111	–.031	–.247	–.063	–.009	–.729**	–.398*	–.242	.627**	.285	.355*	–.467**	–.022	.061	1	.538**	.385*
	Sig. (2-tailed)	.848	.763	.502	.852	.130	.702	.955	.000	.012	.138	.000	.079	.027	.003	.894	.712		.000	.016
	N	39	39	39	39	39	39	39	39	39	39	39	39	39	39	39	39	39	39	39
%switch_LN	Pearson Correlation	–.003	.039	–.070	–.075	–.166	–.336*	–.119	–.609**	–.662**	–.312	.305	.500**	.252	–.204	–.500**	–.180	.538**	1	.411**
	Sig. (2-tailed)	.983	.812	.672	.650	.313	.037	.471	.000	.000	.053	.059	.001	.122	.213	.001	.273	.000		.009
	N	39	39	39	39	39	39	39	39	39	39	39	39	39	39	39	39	39	39	39
%switch_H2	Pearson Correlation	.119	.019	–.393*	–.593**	–.520**	–.529**	–.523**	–.439**	–.474**	–.725**	–.060	–.201	.176	–.325*	–.343*	–.387*	.385*	.411**	1
	Sig. (2-tailed)	.469	.906	.013	.000	.001	.001	.001	.005	.002	.000	.718	.221	.284	.044	.032	.015	.016	.009	
	N	39	39	39	39	39	39	39	39	39	39	39	39	39	39	39	39	39	39	39

Note. H1 = Rater 1; H2 = Rater 2; LN = Lancaster Norms. *Correlation is significant at the .05 level (2-tailed). **Correlation is significant at the .01 level (2-tailed).
