# Supplementary material for: Objective and Subjective Clustering Methods for Verb Fluency Responses From Individuals With Alzheimer's Dementia and Cognitively Healthy Older Adults
Source: Am J Speech Lang Pathol. 2023 Sep 18;32(5 Suppl):2589–601. doi: 10.1044/2023_AJSLP-22-00290 (PMC10721246; doi:10.1044/2023_AJSLP-22-00290)
Supplement: Supplemental Material S2 [file AJSLP-32-2589-s002.pdf]

Supplemental Material S2. Correlations between all variables in cognitively healthy older adults.

		age	education	MMSE	#correct	#cluster_H1	#cluster_LN	#cluster_H2	cluster size_H1	cluster size_LN	cluster size_H2	#switch_H1	#switch_LN	#switch_H2	%cluster_H1	%cluster_LN	%cluster_H2	%switch_H1	%switch_LN	%switch_H2
age	Pearson Correlation	1	−.002	−.678**	−.235	−.187	−.385	−.212	−.085	−.201	−.157	.010	.399	.162	−.089	−.362	−.165	.171	.575**	.271
	Sig. (2-tailed)		.995	.001	.319	.431	.093	.370	.722	.394	.507	.967	.081	.494	.709	.117	.486	.470	.008	.247
	n	20	20	20	20	20	20	20	20	20	20	20	20	20	20	20	20	20	20	20
education	Pearson Correlation	−.002	1	.076	.263	.408	.494*	.376	−.083	−.085	−.237	.061	−.068	.067	.359	.484*	.369	−.079	−.221	−.099
	Sig. (2-tailed)	.995		.750	.262	.074	.027	.102	.727	.721	.315	.797	.777	.779	.120	.031	.109	.741	.349	.677
	n	20	20	20	20	20	20	20	20	20	20	20	20	20	20	20	20	20	20	20
MMSE	Pearson Correlation	−.678**	.076	1	.254	.086	.462*	.199	.063	−.048	−.024	.169	−.139	−.077	−.054	.463*	.149	.034	−.310	−.205
	Sig. (2-tailed)	.001	.750		.281	.719	.040	.401	.793	.842	.920	.477	.559	.746	.822	.040	.531	.886	.183	.385
	n	20	20	20	20	20	20	20	20	20	20	20	20	20	20	20	20	20	20	20
#correct	Pearson Correlation	−.235	.263	.254	1	.579**	.625**	.789**	.189	.226	−.210	.245	.366	.183	.234	.384	.623**	−.284	−.322	−.424
	Sig. (2-tailed)	.319	.262	.281		.008	.003	.000	.425	.339	.374	.297	.112	.439	.322	.095	.003	.225	.166	.063
	n	20	20	20	20	20	20	20	20	20	20	20	20	20	20	20	20	20	20	20
#cluster_H1	Pearson Correlation	−.187	.408	.086	.579**	1	.744**	.772**	−.306	−.092	−.317	.000	.026	.000	.921**	.648**	.740**	−.312	−.330	−.331
	Sig. (2-tailed)	.431	.074	.719	.008		.000	.000	.189	.698	.173	1.000	.913	1.000	.000	.002	.000	.181	.155	.154
	n	20	20	20	20	20	20	20	20	20	20	20	20	20	20	20	20	20	20	20
#cluster_LN	Pearson Correlation	−.385	.494*	.462*	.625**	.744**	1	.748**	−.229	−.246	−.310	.270	−.078	.048	.559*	.955**	.672**	−.070	−.474*	−.321
	Sig. (2-tailed)	.093	.027	.040	.003	.000		.000	.331	.296	.184	.249	.744	.841	.010	.000	.001	.769	.035	.168
	n	20	20	20	20	20	20	20	20	20	20	20	20	20	20	20	20	20	20	20
#cluster_H2	Pearson Correlation	−.212	.376	.199	.789**	.772**	.748**	1	−.192	−.091	−.511*	.303	.298	.187	.559*	.584**	.964**	−.122	−.227	−.305
	Sig. (2-tailed)	.370	.102	.401	.000	.000	.000		.417	.704	.021	.193	.202	.429	.010	.007	.000	.610	.336	.191
	n	20	20	20	20	20	20	20	20	20	20	20	20	20	20	20	20	20	20	20
cluster size_H1	Pearson Correlation	−.085	−.083	.063	.189	−.306	−.229	−.192	1	.721**	.623**	−.645**	−.217	−.448*	−.432	−.309	−.286	−.717**	−.366	−.499*
	Sig. (2-tailed)	.722	.727	.793	.425	.189	.331	.417		.000	.003	.002	.358	.048	.057	.184	.222	.000	.112	.025
	n	20	20	20	20	20	20	20	20	20	20	20	20	20	20	20	20	20	20	20
cluster size_LN	Pearson Correlation	−.201	−.085	−.048	.226	−.092	−.246	−.091	.721**	1	.476*	−.533*	−.406	−.267	−.172	−.347	−.151	−.667**	−.609**	−.389
	Sig. (2-tailed)	.394	.721	.842	.339	.698	.296	.704	.000		.034	.015	.076	.256	.468	.134	.525	.001	.004	.090
	n	20	20	20	20	20	20	20	20	20	20	20	20	20	20	20	20	20	20	20
cluster size_H2	Pearson Correlation	−.157	−.237	−.024	−.210	−.317	−.310	−.511*	.623**	.476*	1	−.661**	−.462*	−.767**	−.272	−.285	−.587**	−.555*	−.316	−.580**
	Sig. (2-tailed)	.507	.315	.920	.374	.173	.184	.021	.003	.034		.001	.040	.000	.245	.224	.006	.011	.175	.007
	n	20	20	20	20	20	20	20	20	20	20	20	20	20	20	20	20	20	20	20
#switch_H1	Pearson Correlation	.010	.061	.169	.245	.000	.270	.303	−.645**	−.533*	−.661**	1	.524*	.739**	−.136	.224	.270	.852**	.350	.505*
	Sig. (2-tailed)	.967	.797	.477	.297	1.000	.249	.193	.002	.015	.001		.018	.000	.567	.343	.250	.000	.130	.023
	n	20	20	20	20	20	20	20	20	20	20	20	20	20	20	20	20	20	20	20
#switch_LN	Pearson Correlation	.399	−.068	−.139	.366	.026	−.078	.298	−.217	−.406	−.462*	.524*	1	.471*	−.115	−.236	.251	.348	.752**	.206
	Sig. (2-tailed)	.081	.777	.559	.112	.913	.744	.202	.358	.076	.040	.018		.036	.629	.316	.287	.133	.000	.384
	n	20	20	20	20	20	20	20	20	20	20	20	20	20	20	20	20	20	20	20
#switch_H2	Pearson Correlation	.162	.067	−.077	.183	.000	.048	.187	−.448*	−.267	−.767**	.739**	.471*	1	−.095	−.013	.155	.642**	.327	.805**
	Sig. (2-tailed)	.494	.779	.746	.439	1.000	.841	.429	.048	.256	.000	.000	.036		.691	.956	.514	.002	.159	.000
	n	20	20	20	20	20	20	20	20	20	20	20	20	20	20	20	20	20	20	20
%cluster_H1	Pearson Correlation	−.089	.359	−.054	.234	.921**	.559*	.559*	−.432	−.172	−.272	−.136	−.115	−.095	1	.545*	.604**	−.273	−.226	−.219
	Sig. (2-tailed)	.709	.120	.822	.322	.000	.010	.010	.057	.468	.245	.567	.629	.691		.013	.005	.244	.337	.354
	n	20	20	20	20	20	20	20	20	20	20	20	20	20	20	20	20	20	20	20
%cluster_LN	Pearson Correlation	−.362	.484*	.463*	.384	.648**	.955**	.584**	−.309	−.347	−.285	.224	−.236	−.013	.545*	1	.557*	.006	−.473*	−.236
	Sig. (2-tailed)	.117	.031	.040	.095	.002	.000	.007	.184	.134	.224	.343	.316	.956	.013		.011	.978	.035	.317
	n	20	20	20	20	20	20	20	20	20	20	20	20	20	20	20	20	20	20	20
%cluster_H2	Pearson Correlation	−.165	.369	.149	.623**	.740**	.672**	.964**	−.286	−.151	−.587**	.270	.251	.155	.604**	.557*	1	−.073	−.166	−.243
	Sig. (2-tailed)	.486	.109	.531	.003	.000	.001	.000	.222	.525	.006	.250	.287	.514	.005	.011		.759	.483	.302
	n	20	20	20	20	20	20	20	20	20	20	20	20	20	20	20	20	20	20	20
%switch_H1	Pearson Correlation	.171	−.079	.034	−.284	−.312	−.070	−.122	−.717**	−.667**	−.555*	.852**	.348	.642**	−.273	.006	−.073	1	.547*	.741**
	Sig. (2-tailed)	.470	.741	.886	.225	.181	.769	.610	.000	.001	.011	.000	.133	.002	.244	.978	.759		.013	.000
	n	20	20	20	20	20	20	20	20	20	20	20	20	20	20	20	20	20	20	20
%switch_LN	Pearson Correlation	.575**	−.221	−.310	−.322	−.330	−.474*	−.227	−.366	−.609**	−.316	.350	.752**	.327	−.226	−.473*	−.166	.547*	1	.489*
	Sig. (2-tailed)	.008	.349	.183	.166	.155	.035	.336	.112	.004	.175	.130	.000	.159	.337	.035	.483	.013		.029
	n	20	20	20	20	20	20	20	20	20	20	20	20	20	20	20	20	20	20	20
%switch_H2	Pearson Correlation	.271	−.099	−.205	−.424	−.331	−.321	−.305	−.499*	−.389	−.580**	.505*	.206	.805**	−.219	−.236	−.243	.741**	.489*	1
	Sig. (2-tailed)	.247	.677	.385	.063	.154	.168	.191	.025	.090	.007	.023	.384	.000	.354	.317	.302	.000	.029	
	n	20	20	20	20	20	20	20	20	20	20	20	20	20	20	20	20	20	20	20

Note. H1 = Rater 1; H2 = Rater 2; LN = Lancaster Norms. *Correlation is significant at the .05 level (2-tailed). **Correlation is significant at the .01 level (2-tailed).
