# Supplementary material for: Objective and Subjective Clustering Methods for Verb Fluency Responses From Individuals With Alzheimer's Dementia and Cognitively Healthy Older Adults
Source: Am J Speech Lang Pathol. 2023 Sep 18;32(5 Suppl):2589–601. doi: 10.1044/2023_AJSLP-22-00290 (PMC10721246; doi:10.1044/2023_AJSLP-22-00290)
Supplement: Supplemental Material S3 [file AJSLP-32-2589-s003.pdf]

Supplemental material, Fisher et al., "Objective and subjective clustering methods for verb fluency responses from individuals with Alzheimer's dementia and cognitively healthy older adults," *AJSLP*, https://doi.org/10.1044/2023_AJSLP-22-00290

Supplemental Material S3. Correlations between all variables in participants with Alzheimer's Disease.

		age	education	MMSE	#correct	#cluster_H1	#cluster_LN	#cluster_H2	cluster size_H1	cluster size_LN	cluster size_H2	#switch_H1	#switch_LN	#switch_H2	%cluster_H1	%cluster_LN	%cluster_H2	%switch_H1	%switch_LN	%switch_H2
age	Pearson Correlation	1	–.153	–.077	.292	.297	.493*	.325	.296	.301	.355	.141	.039	.166	.200	.535*	.317	–.097	–.402	–.318
	Sig. (2-tailed)		.531	.753	.224	.217	.032	.174	.219	.210	.136	.564	.875	.496	.413	.018	.185	.694	.088	.185
	n	19	19	19	19	19	19	19	19	19	19	19	19	19	19	19	19	19	19	19
education	Pearson Correlation	–.153	1	–.276	–.355	–.376	–.374	–.392	–.333	–.338	–.375	–.239	–.220	–.337	–.456*	–.433	–.456*	.146	.229	.175
	Sig. (2-tailed)	.531		.253	.136	.112	.115	.097	.163	.157	.114	.325	.365	.158	.050	.064	.050	.551	.346	.474
	n	19	19	19	19	19	19	19	19	19	19	19	19	19	19	19	19	19	19	19
MMSE	Pearson Correlation	–.077	–.276	1	.411	.260	.307	.189	.323	.190	.502*	.250	.314	.323	.190	.188	.115	–.131	–.064	–.236
	Sig. (2-tailed)	.753	.253		.080	.282	.201	.437	.177	.436	.028	.303	.191	.177	.436	.440	.640	.592	.794	.332
	n	19	19	19	19	19	19	19	19	19	19	19	19	19	19	19	19	19	19	19
#correct	Pearson Correlation	.292	–.355	.411	1	.821**	.779**	.842**	.188	.435	.725**	.817**	.839**	.833**	.360	.429	.512*	.147	–.031	–.512*
	Sig. (2-tailed)	.224	.136	.080		.000	.000	.000	.440	.063	.000	.000	.000	.000	.130	.067	.025	.548	.899	.025
	n	19	19	19	19	19	19	19	19	19	19	19	19	19	19	19	19	19	19	19
#cluster_H1	Pearson Correlation	.297	–.376	.260	.821**	1	.774**	.751**	.255	.343	.563*	.449	.631**	.658**	.757**	.472*	.495*	–.219	–.122	–.438
	Sig. (2-tailed)	.217	.112	.282	.000		.000	.000	.293	.150	.012	.054	.004	.002	.000	.041	.031	.367	.620	.061
	n	19	19	19	19	19	19	19	19	19	19	19	19	19	19	19	19	19	19	19
#cluster_LN	Pearson Correlation	.493*	–.374	.307	.779**	.774**	1	.710**	.308	.281	.688**	.487*	.485*	.562*	.463*	.839**	.522*	–.021	–.317	–.557*
	Sig. (2-tailed)	.032	.115	.201	.000	.000		.001	.200	.244	.001	.034	.035	.012	.046	.000	.022	.931	.186	.013
	n	19	19	19	19	19	19	19	19	19	19	19	19	19	19	19	19	19	19	19
#cluster_H2	Pearson Correlation	.325	–.392	.189	.842**	.751**	.710**	1	.120	.484*	.540*	.734**	.662**	.651**	.446	.488*	.850**	.186	–.096	–.527*
	Sig. (2-tailed)	.174	.097	.437	.000	.000	.001		.625	.036	.017	.000	.002	.003	.056	.034	.000	.445	.694	.020
	n	19	19	19	19	19	19	19	19	19	19	19	19	19	19	19	19	19	19	19
cluster size_H1	Pearson Correlation	.296	–.333	.323	.188	.255	.308	.120	1	.668**	.405	–.239	–.217	.034	.406	.361	.146	–.749**	–.814**	–.339
	Sig. (2-tailed)	.219	.163	.177	.440	.293	.200	.625		.002	.085	.325	.373	.890	.084	.129	.552	.000	.000	.156
	n	19	19	19	19	19	19	19	19	19	19	19	19	19	19	19	19	19	19	19
cluster size_LN	Pearson Correlation	.301	–.338	.190	.435	.343	.281	.484*	.668**	1	.510*	.230	.022	.227	.254	.303	.460*	–.256	–.722**	–.498*
	Sig. (2-tailed)	.210	.157	.436	.063	.150	.244	.036	.002		.026	.344	.927	.351	.294	.207	.048	.289	.000	.030
	n	19	19	19	19	19	19	19	19	19	19	19	19	19	19	19	19	19	19	19
cluster size_H2	Pearson Correlation	.355	–.375	.502*	.725**	.563*	.688**	.540*	.405	.510*	1	.463*	.381	.362	.262	.549*	.388	–.008	–.372	–.789**
	Sig. (2-tailed)	.136	.114	.028	.000	.012	.001	.017	.085	.026		.046	.108	.127	.279	.015	.100	.973	.117	.000
	n	19	19	19	19	19	19	19	19	19	19	19	19	19	19	19	19	19	19	19
#switch_H1	Pearson Correlation	.141	–.239	.250	.817**	.449	.487*	.734**	–.239	.230	.463*	1	.875**	.816**	–.027	.239	.490*	.635**	.308	–.230
	Sig. (2-tailed)	.564	.325	.303	.000	.054	.034	.000	.325	.344	.046		.000	.000	.914	.325	.033	.004	.199	.344
	n	19	19	19	19	19	19	19	19	19	19	19	19	19	19	19	19	19	19	19
#switch_LN	Pearson Correlation	.039	–.220	.314	.839**	.631**	.485*	.662**	–.217	.022	.381	.875**	1	.907**	.197	.110	.346	.401	.484*	–.082
	Sig. (2-tailed)	.875	.365	.191	.000	.004	.035	.002	.373	.927	.108	.000		.000	.419	.654	.147	.089	.036	.740
	n	19	19	19	19	19	19	19	19	19	19	19	19	19	19	19	19	19	19	19
#switch_H2	Pearson Correlation	.166	–.337	.323	.833**	.658**	.562*	.651**	.034	.227	.362	.816**	.907**	1	.271	.284	.400	.263	.219	.039
	Sig. (2-tailed)	.496	.158	.177	.000	.002	.012	.003	.890	.351	.127	.000	.000		.261	.239	.090	.277	.367	.874
	n	19	19	19	19	19	19	19	19	19	19	19	19	19	19	19	19	19	19	19
%cluster_H1	Pearson Correlation	.200	–.456*	.190	.360	.757**	.463*	.446	.406	.254	.262	–.027	.197	.271	1	.400	.424	–.596**	–.223	–.227
	Sig. (2-tailed)	.413	.050	.436	.130	.000	.046	.056	.084	.294	.279	.914	.419	.261		.090	.070	.007	.359	.349
	n	19	19	19	19	19	19	19	19	19	19	19	19	19	19	19	19	19	19	19
%cluster_LN	Pearson Correlation	.535*	–.433	.188	.429	.472*	.839**	.488*	.361	.303	.549*	.239	.110	.284	.400	1	.567*	–.025	–.525*	–.419
	Sig. (2-tailed)	.018	.064	.440	.067	.041	.000	.034	.129	.207	.015	.325	.654	.239	.090		.011	.918	.021	.074
	n	19	19	19	19	19	19	19	19	19	19	19	19	19	19	19	19	19	19	19
%cluster_H2	Pearson Correlation	.317	–.456*	.115	.512*	.495*	.522*	.850**	.146	.460*	.388	.490*	.346	.400	.424	.567*	1	.182	–.216	–.370
	Sig. (2-tailed)	.185	.050	.640	.025	.031	.022	.000	.552	.048	.100	.033	.147	.090	.070	.011		.457	.375	.119
	n	19	19	19	19	19	19	19	19	19	19	19	19	19	19	19	19	19	19	19
%switch_H1	Pearson Correlation	–.097	.146	–.131	.147	–.219	–.021	.186	–.749**	–.256	–.008	.635**	.401	.263	–.596**	–.025	.182	1	.537*	.121
	Sig. (2-tailed)	.694	.551	.592	.548	.367	.931	.445	.000	.289	.973	.004	.089	.277	.007	.918	.457		.018	.623
	n	19	19	19	19	19	19	19	19	19	19	19	19	19	19	19	19	19	19	19
%switch_LN	Pearson Correlation	–.402	.229	–.064	–.031	–.122	–.317	–.096	–.814**	–.722**	–.372	.308	.484*	.219	–.223	–.525*	–.216	.537*	1	.472*
	Sig. (2-tailed)	.088	.346	.794	.899	.620	.186	.694	.000	.000	.117	.199	.036	.367	.359	.021	.375	.018		.041
	n	19	19	19	19	19	19	19	19	19	19	19	19	19	19	19	19	19	19	19
%switch_H2	Pearson Correlation	–.318	.175	–.236	–.512*	–.438	–.557*	–.527*	–.339	–.498*	–.789**	–.230	–.082	.039	–.227	–.419	–.370	.121	.472*	1
	Sig. (2-tailed)	.185	.474	.332	.025	.061	.013	.020	.156	.030	.000	.344	.740	.874	.349	.074	.119	.623	.041	
	n	19	19	19	19	19	19	19	19	19	19	19	19	19	19	19	19	19	19	19

Note. H1 = Rater 1; H2 = Rater 2; LN = Lancaster Norms; size = average cluster size. *Correlation is significant at the .05 level (2-tailed). **Correlation is significant at the .01 level (2-tailed).
